# Kidney Tumor Segmentation Based on FR2PAttU-Net Model

**DOI:** 10.3389/fonc.2022.853281

**Published:** 2022-03-17

**Authors:** Peng Sun, Zengnan Mo, Fangrong Hu, Fang Liu, Taiping Mo, Yewei Zhang, Zhencheng Chen

**Affiliations:** ^1^ School of Electronic Engineering and Automation, Guilin University of Electronic Technology, Guilin, China; ^2^ Center for Genomic and Personalized Medicine, Guangxi Medical University, Nanning, China; ^3^ College of Life and Environment Science, Guilin University of Electronic Technology, Guilin, China; ^4^ Hepatopancreatobiliary Center, The Second Affiliated Hospital of Nanjing Medical University, Nanjing, China

**Keywords:** kidney tumor segmentation, FR2PAttU-Net, KiTS19, data augmentation, CT

## Abstract

The incidence rate of kidney tumors increases year by year, especially for some incidental small tumors. It is challenging for doctors to segment kidney tumors from kidney CT images. Therefore, this paper proposes a deep learning model based on FR2PAttU-Net to help doctors process many CT images quickly and efficiently and save medical resources. FR2PAttU-Net is not a new CNN structure but focuses on improving the segmentation effect of kidney tumors, even when the kidney tumors are not clear. Firstly, we use the R2Att network in the “U” structure of the original U-Net, add parallel convolution, and construct FR2PAttU-Net model, to increase the width of the model, improve the adaptability of the model to the features of different scales of the image, and avoid the failure of network deepening to learn valuable features. Then, we use the fuzzy set enhancement algorithm to enhance the input image and construct the FR2PAttU-Net model to make the image obtain more prominent features to adapt to the model. Finally, we used the KiTS19 data set and took the size of the kidney tumor as the category judgment standard to enhance the small sample data set to balance the sample data set. We tested the segmentation effect of the model at different convolution and depths, and we got scored a 0.948 kidney Dice and a 0.911 tumor Dice results in a 0.930 composite score, showing a good segmentation effect.

## Introduction

In recent years, the incidence rate of kidney tumors has increased (1–3). If we rely on artificial ways to process medical image data of patients, it will waste a lot of time. And because of the difference in medical experience, some small and challenging methods to find tumors are easily ignored by doctors, and subjective factors lead to misjudgment. Therefore, how to use the deep learning model to segment kidney tumors is a challenging task (4). However, most kidney image analysis is usually based on kidney segmentation rather than tumor segmentation or two deep models: the first to segment the kidney and the second to segment the tumor on the kidney (5, 6). Among many current research schemes, they get scored about 0.97 kidney Dice and 0.85 tumor Dice (7). These methods can provide higher values from the extracted features by pre-analyzing the information provided by the image; they play a role in the early detection and diagnosis of abnormalities. However, new research in this field is still significant because effective and accurate segmentation always has room for improvement, especially considering ignoring minor medical errors (8, 9). In these cases, the segmentation task of kidney and kidney tumors becomes more complex (10). Therefore, it is necessary to study the application of more in-depth learning methods in kidney tumors without manual intervention, improve the analysis efficiency, and reduce workload of experts to improve the segmentation effect of tumors.

This paper proposes an automatic segmentation method of kidney and tumor in CT image to support the diagnosis of kidney disease of experts: a flexible model that can segment kidneys and tumors simultaneously. In the design of our improved model, we consider the primary shortcomings of the existing deep learning model and develop a new, efficient and automatic kidney segmentation method. In this article, we emphasize the following contributions:

(1) We use the cascade network model. The first model is used to coarse segment the kidney and tumor ROI (the kidney without tumor is not segmented). The second model is used to finely segment the tumor in CT images to improve the segmentation effect of the tumor.
(2) We propose to reconstruct labeled CT images based on tumor size to balance the kidney tumor data set and reduce the impact of category imbalance.
(3) We propose the FR2PAttU-Net model and verify it in the KiTS19 data set. Finally, it can segment tumors with high precision, even when kidney tumors are unclear.

Therefore, we believe that the proposed FR2PAttU-Net model provides an effective kidney tumor segmentation method, improving the segmentation effect and diagnosis rate of kidney tumors.

The overall structure of this paper is as follows. Section 2 introduces the relevant research and findings; Section 3 discusses the methods; Section 4 reports the experiments carried out to verify our research, the comparative analysis of the corresponding results and other similar studies, and Section 5 gives the discussion and conclusions.

### Related Work

The task of kidney segmentation has not only recently started. Several methods have been developed in the past few years, and more and more expressive results have been obtained to solve this problem.

In 2015, Ronneberger et al. (11) proposed the U-Net model to realize the segmentation of medical images. The U-Net model is one of the earliest algorithms for semantic segmentation using a Fully Convolutional Network. The symmetric U-shaped structure that contains the compression path and the expansion path in the paper was very innovative at the time. Due to its relatively simple task, U-Net has achieved a meager error rate through 30 pictures, supplemented by a data expansion strategy, and won the championship’s championship. First, it established the position of the U-Net model in medical image segmentation. Then a variant algorithm based on the U-Net model is applied in multiple directions of medical image segmentation.

Since U-Net, a series of algorithms have been derived for medical image segmentation. For example, Yang et al. (12) proposed a method for measuring lung parenchymal parameters based on the ResU-Net model based on lung window CT images, and analyzed the relationship between lung volume and CT value or density, and concluded that lung volume is negatively correlated with CT value or density. Oktay et al. (13) proposed a new attention gate (AG) model for medical imaging, which can automatically learn to focus on target structures of different shapes and sizes, and use the model trained by AGs to implicitly learn to suppress outside areas in the input image while highlighting salient features useful for specific tasks. The experimental results show that, while maintaining computational efficiency, AGs consistently improve the prediction performance of U-Net under different data sets and training scales. Alom et al. (14) proposed a U-Net-based recurrent convolutional neural network (RCNN) and a U-Net model-based recurrent, residual convolutional neural network (RRCNN), named RU-Net and R2U-Net, respectively. The proposed model utilizes the capabilities of the U network, residual network, and RCNN. The experimental results show that compared with the equivalent model, including U-Net and residual U-Net (ResU-Net), the model has the advantages of segmentation tasks. Better performance. Wang et al. (15) used U-net combined with the recurrent residual and attention models to segment the image. Experiments show that they can obtain better results.

Since 2020, the segmentation of kidney and kidney tumors based on the U-Net model has gradually increased. Isensee et al. (16) introduced nnU-Net (‘no-new-Net’), which eliminated many of the powerful reasons for the unnecessary bells and whistles in the proposed network design, and instead focused on the remaining aspects of the performance and versatility of the composition method. nnU-Net achieved the highest average dice score in the challenge online leaderboard. Da Cruz et al. (17) used U-Net 2D for initial segmentation and delineated the kidney (CT) image. In the KiTS19 challenge, its average Dice coefficient is 93.03%. Turk et al. (18) used the superior characteristics of the existing V-Net model to propose a new hybrid model, which improved the previously unapplied encoder and decoder stages and obtained 97.7% kidney Dice and 86.5% tumor Dice.

In 2021, Heller et al. (19) released the KiTS19 challenge and published the top five methods and segmentation effects in the article: The fifth place was made by Ma (20). A 3D U-Net is used as the main architecture which is based on nnU-Net implementation. Compared to the original 3D U-Net, the notable changes are padding convolutions, instance normalization, and leaky-ReLUs. This submission scored a 0.973 kidney Dice, and a 0.825 tumor Dice resulting in a 0.899 composite score. The fourth place was made by Hou et al. (21). They use a cascaded volumetric convolutional network for kidney tumor segmentation from CT volumes. There are two steps in this model, and one is coarse location, the other is fine predictions. This submission scored a 0.974 kidney Dice and a 0.831 tumor Dice resulting in a 0.902 composite score. The third place was made by Mu et al. (22). They used multi-resolution VB-nets for segmentation of kidney tumor, and they scored a 0.973 kidney Dice and a 0.832 tumor Dice resulting in a 0.903 composite score. The second place was made by Hou et al. (23). They used cascaded semantic segmentation for kidney and tumor. This cascaded approach had three stages. Stage 1 performed a coarse segmentation of all kidneys in the image. The second stage is run for each rectangular kidney region that is found by the first stage, and in the third stage of the model, a fully convolutional net is used to segment the tumor voxels from the kidney voxels. This submission scored a 0.967 kidney Dice and a 0.845 tumor Dice resulting in a 0.906 composite score. The first place was made by Isensee et al. (24). Three 3D U-Net architectures were tested using five-fold cross validation, and this submission scored a 0.974 kidney Dice and a 0.851 tumor Dice resulting in a 0.912 composite score.

Based on the above analysis, we find that most algorithms in the field of medical image segmentation take the U-Net architecture as the starting point for further development and derive a series of improved and variant algorithms from realizing the task of medical image segmentation. Although most models can achieve good results, there is always room for effective and accurate segmentation improvement. Furthermore, although multiple networks will increase the time cost, they can improve the segmentation effect simultaneously. Therefore, in this work, we propose the FR2PAttU-Net model to improve the segmentation performance of kidney tumor CT images.

## Materials and Methods

This section will introduce the overall scheme of kidney tumor segmentation. The first section introduces the structure of the FR2PAttU-Net model for kidney and tumor segmentation. The second section presents the steps of kidney tumor segmentation, namely, data preparation, coarse segmentation, and fine segmentation. We will explain each piece in detail next.

### FR2PAttU-Net

**Figure 1:**
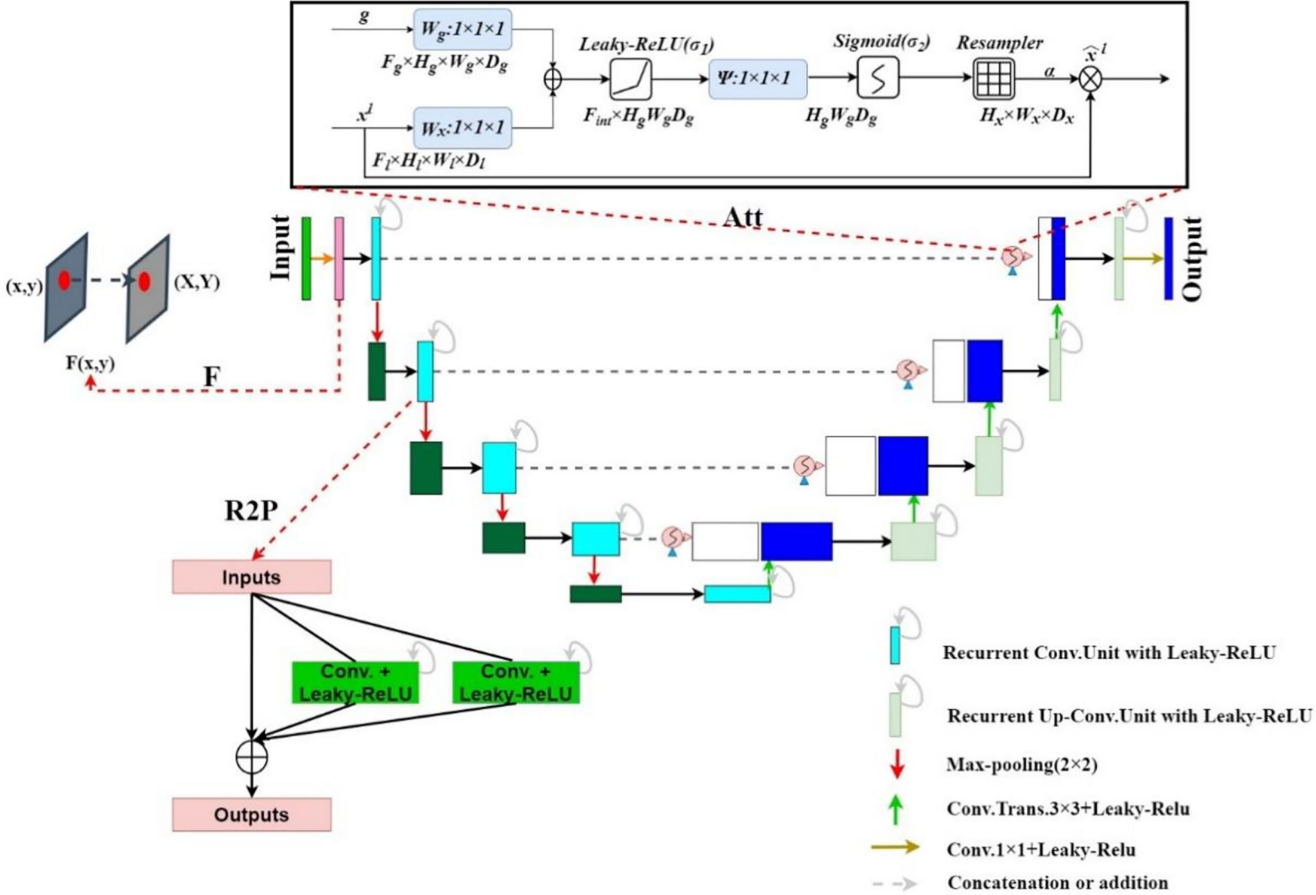
FR2PAttU-Net Model.

We propose the FR2PAttU-Net model, where F, R2, P, and Att are the abbreviations for Fuzzy set, Recurrent Residual, Parallel, and Attention, respectively. The “U”-shaped architecture of the standard U-Net is used in our network. **Figure 1** shows the architecture and layers that make up our network, with the contraction path defined on the left of the model and the symmetrical expansion path specified on the right. All convolutional layers are modified from consecutive 3 × 3 kernels to parallel kernels, and we will introduce the specific structures and functions of F, R2, P, and Att step by step. Furthermore, we use the activation function Leaky-ReLU.

#### Image Enhancement Based on Fuzzy Set (F)

Image enhancement emphasizes or sharpens certain features of an image, such as edges, contours, contrast, etc., for display, observation, or further analysis and processing. The processed image is transformed through specific image processing into an image of better visual quality and effect or more “useful” for a particular application. Fuzzy sets provide a form of loose processing information. For example, using fuzzy sets to enhance images of kidneys and kidney tumors can make the entire kidney more clearly delineated, making it more adaptable to the network.

Image enhancement based on fuzzy sets mainly includes three steps: image fuzzy feature extraction, membership function value correction, and fuzzy domain inverse transformation (25). Define Z as an object set, where z represents a type of element in Z. A fuzzy set A in Z is mainly characterized by a degree of membership *μ_A_(z)*. In this regard, the fuzzy set A is composed of z-values and membership

We use fuzzy sets to perform a gray-scale transformation to enhance the image. Then, we stipulate the following fuzzy rules:

R1: IF one pixel is dark, THEN makes this pixel darker;

R2: IF one pixel is gray, THEN keeps it gray;

R3: IF one pixel is bright, THEN makes this pixel brighter;

This rule represents our approach. But, of course, the pixels in the IF condition are dark (either gray or bright), and this concept is blurred. In the same way, the darker (or staying gray, or merrier) in the THEN conclusion is also fuzzy. To this end, we need to establish a membership function to determine the membership of a pixel to three conditions (26).

The determination of the membership function is very complicated. However, here we try to make it simple. First, a pixel is dark (fuzzy), then the approximate shape of its membership function is that the domain membership is 1 when it is lower than a certain value z1. After the gray level crosses a specific value, z2, its membership degree is 0. So, of course, z1 ≠ z2. Then we perform linear interpolation between z1 and z2, and then we can get the membership function of R1. Similarly, R2 and R3 are the same.

For pixel Z_0_, it is necessary to calculate the corresponding membership degrees μ_dark_(Z_0_), *μ*_gray_(Z_0_), and *μ*_bright_(Z_0_) according to the rules R1, R2, and R3. This process is called fuzzification. The function (or the corresponding relationship) used to fuzz an input quantity is the knowledge base.

After fuzzification, the three membership degrees μ_dark_(Z_0_), μ_gray_(Z_0_), and μ_bright_(Z_0_) corresponding to a pixel can be deblurred. There are many de-obfuscation algorithms, and Equation (1) is the center of gravity method.


(1) $$v_{0}=\frac{\mu_{dark}(z_{0})\times v_{d}+\mu_{gray}(z_{0})\times v_{g}+\mu_{bright}(z_{0})\times v_{b}}{\mu_{dark}(z_{0})+\mu_{gray}(z_{0})+\mu_{bright}(z_{0})}$$

Among them, *ν*_d_, *ν_g_*, and ν_b_ are the single output values. Then, pixel Z_0_ must calculate the corresponding membership degrees μ_dark_(Z_0_), μ_gray_(Z_0_), and μ_bright_(Z_0_) according to R1, R2, and R3. Finally, we obtain a weighted maturity estimate, which is the most output value. At this point, the output *ν*_0_ is obtained.

The specific transformation result can be obtained by Equations (2) and (3).


(2) m=image[x][y]

(3) $$f(x)=\{\begin{array}{r} 0,\;0\leq m<0.15\\ (m-0.15)/0.28\times 127,\;015\leq x<0.43\\ (m-0.45)/0.28\times 255+(0.71-m)/0.28\times 127,\;0.43\leq x<0.71\\ 255,else \end{array}$$

*image*[*x*][*y*] is the pixel value at point (x, y), This article takes m values 0.15, 0.43, 0.71, 1, respectively, and divides the entire pixel value into four regions to complete the pixel conversion.

**Figure 2:**
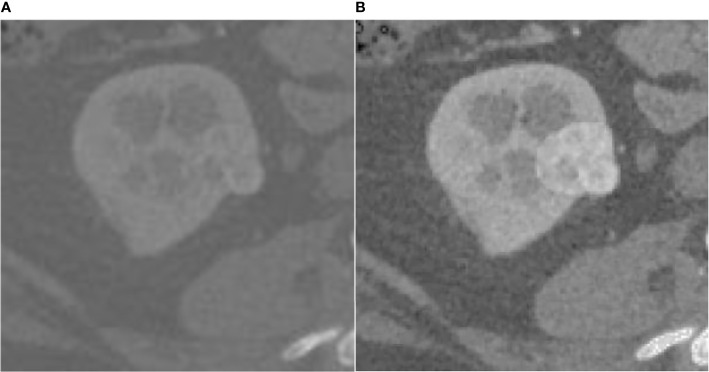
Result of fuzz set enhancement algorithm, **(A)** is the original CT image, **(B)** is the image enhanced by the fuzzy set.

The effect of the fuzzy set enhancement algorithm is shown in the **Figure 2**.

#### Recurrent-Residual-Parallel Convolutional Network (R2P)

**Figure 3:**
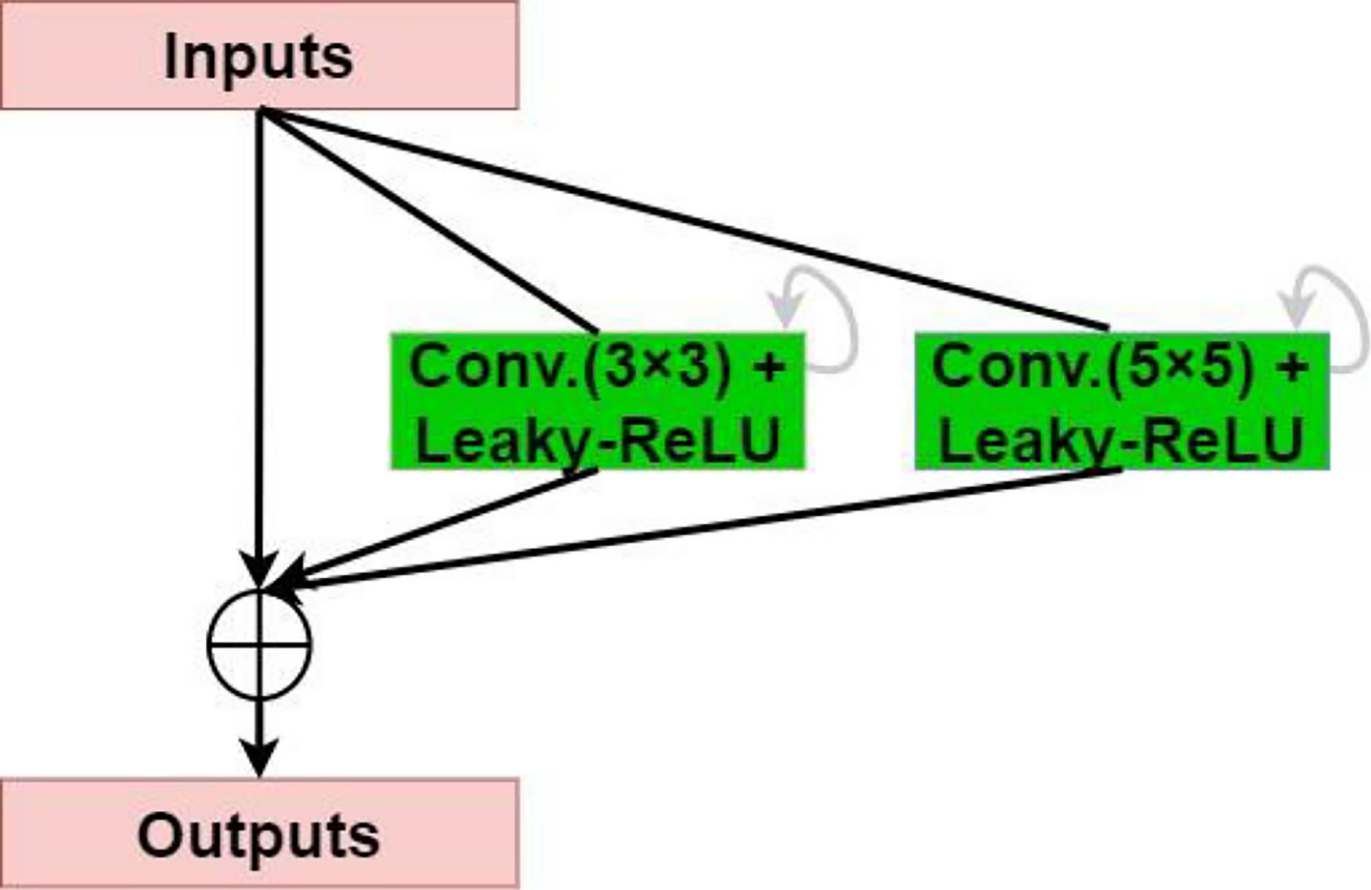
Recurrent-residual-parallel convolutional network (R2P).

The residual network enables the training of deeper networks, and the recurrent residual convolutional layer allows the network to extract better features. The network provides for the network to deepen and avoid the inability to learn the gradient under the same amount of parameters, resulting in better performance. As shown in **Figure 3**, the model uses the recurrent residual block instead of the traditional Conv + ReLU layer in the encoding and decoding process, which can train a deeper network. All convolution layers are composed of successive convolution (convolution kernel 3 × 3) are modified to parallel convolutional network, and we tested parallel convolutions (convolution kernel = 3 × 3), and parallel convolutions (convolution kernel = 3 × 3 and 5 × 5), and perform parallel convolution operations on the image, stitching all outputs into one deep feature map. Different convolution and pooling operations can obtain more information about the input image, and processing these operations in parallel and combining all the results will yield a better image representation. We use different convolution kernels for image feature extraction, which fully increases width of the model, increases the receptive field, and improves the robustness of the network, thereby improving the ability of the model to adapt to features of different scales in the image. Then, summation of features at other time steps is used to obtain a more expressive quality, which helps extract lower-level features; finally, skip connections are not cut off in the original U-Net but are cascaded operate.

#### Attention Gate (Att)

**Figure 4:**
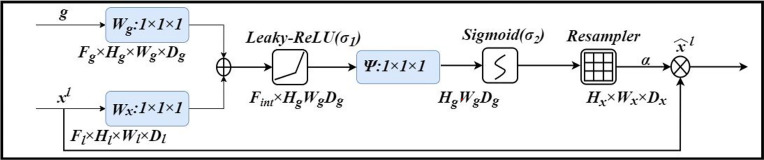
Attention Gate (Att).

An attention gate is added to the model, which automatically learns to distinguish the shape and size of objects. **Figure 4** shows the calculation method of the attention gate. First, the output g corresponding to the decoder part is upsampled + convolved, and then 1-dimensional convolution is used to reduce the dimension of g and x (from the encoder at the same level). As a result, the number of channels becomes 1/2 of the original. Then the two parts of the results are added; after the activation function Leaky-ReLU and one-dimensional convolution, the number of channels is reduced to 1. Then through the Sigmod function, a 1-dimensional attention map with the same size as x is obtained, and the original x is used as element-wise multiplication to get a weighted vector.

#### Leaky-ReLU

Furthermore, the Leaky-ReLU activation function and batch normalization follow closely (27). The difference from ReLU is that the negative axis of Leaky-ReLU retains a tiny constant leak so that when the input information is less than 0, the information is not wholly lost, and the corresponding retention is carried out. That is, ReLU has no gradient when the value is less than zero, and Leaky-ReLU gives a slight incline when the value is less than 0. It is equivalent to allowing backpropagation of gradients corresponding to intervals less than 0 rather than direct interception.

### Segmentation Scheme

**Figure 5:**
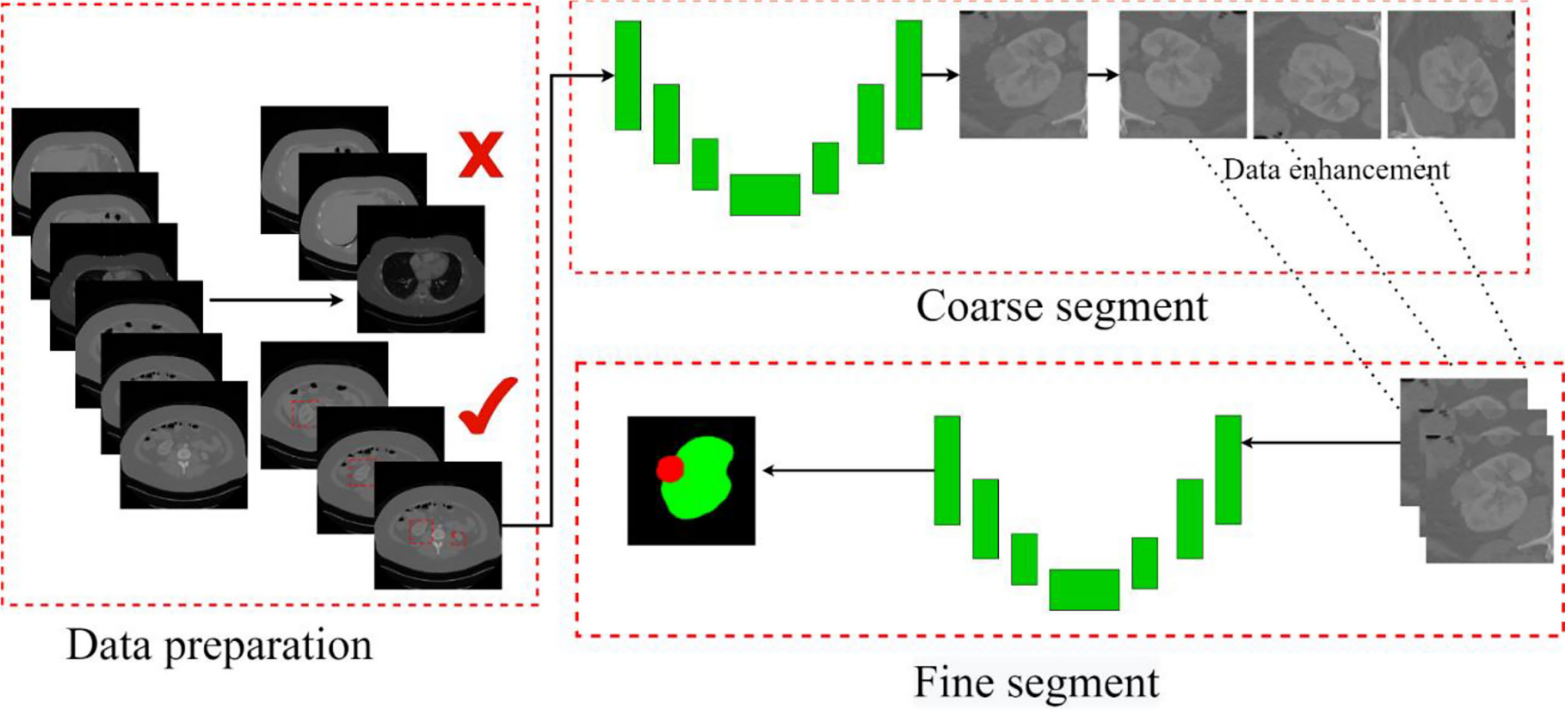
The overall process. Contains three parts: data preparation, coarse segmentation, and fine segmentation. Section *Data Preparation* introduces data preparation in detail, Section *Coarse Segmentation Based on FR2PAttU-Net Model* introduces coarse segmentation and Section *Fine Segmentation Based on FR2PAttU-Net Model* introduces fine segmentation.

This paper mainly segments kidneys and tumors from three parts. In the first part, kidney data is collected and preprocessed. We picked out the slice range containing the kidney from the CT images and discarded the invalid area that did not include the kidney and tumor. The second part is coarse segmentation. We use the first model to segment the approximate size of the kidney and tumor. This step is only used to locate the initial location of the kidney and tumor, select the ROI, and do not segment. The third part analyzes ROIs and reconstructs CT images with labels to balance the kidney tumor segmentation dataset. Then we use the second model for fine segmentation of kidneys and tumors, where the ROI region is used as the input image to improve the segmentation effect. The segmentation scheme is shown in **Figure 5**. Each of these steps is described in detail in the subsections that follow.

#### Data Preparation

In this study, we downloaded the available data set from the homepage of the KiTS19 data set and did not use additional data. A total of 210 scans with high-quality ground truth segmentations were downloaded from the KiTS19 data set, publicly available on GitHub (https://github.com/neheller/kits19). The homepage of the KiTS19 data set provides other instructions on the preparation of the data set and the ethics committee (28). Manual segmentation may cause many errors in subsequent kidney or kidney tumor monitoring. In addition, it is very time-consuming and may degrade system performance (29). Despite these adverse effects, we still used the KiTS19 dataset because of the lack of available datasets in the literature. Patients with cysts and tumor thrombi were excluded from the KiTS19 dataset because in these patients, the tumor was beyond what we thought was the primary site and the appropriate boundaries were unclear. Therefore, we only selected kidneys with tumor lesions in this study to construct training and test datasets. The task is the segmentation of kidneys and kidney tumors in contrast-enhanced abdominal CT without judging the type of tumor.

To make the data satisfy our network model, we cut the 3D data into several 2D images with 512 × 512 pixels. In addition, all the slices without kidney markers are discarded. Processing the original CT images before sending them to the network is a crucial step for practical training. The first aspect to consider is the presence of unexpected substances that may appear in the body of the patient. In particular, the metal artifacts have a significant negative impact on the quality of CT images, which is a well-known fact. The main problem with artifacts is that the areas generated in the image have abnormal intensity values or are much higher or lower than the intensity values of pixels corresponding to organic tissues. Since deep learning algorithms are based on data-driven models, abnormal voxels corresponding to non-organic artifacts can significantly affect learning. To reduce the impact of non-organic artifacts, we uniformly process the complete data set, namely, training and test data. We only consider the effective intensity range between 0.5 and 99.5% in all images and tailor the outliers accordingly. After preprocessing, data is normalized with the normal foreground mean and standard deviation to improve the training effect of the network.

#### Coarse Segmentation Based on FR2PAttU-Net Model

**Figure 6:**
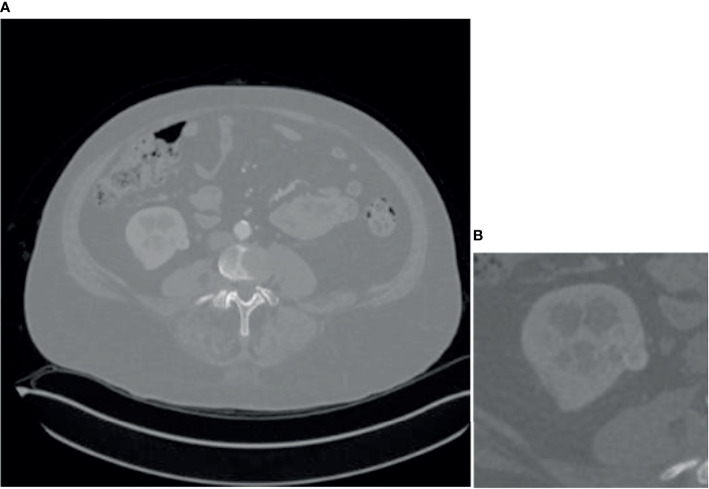
Result of coarse segmentation on CT image of 512 × 512 pixels to 128 × 128 pixels, **(A)** is CT image of 512 × 512 pixels, **(B)** is CT image of 128 × 128 pixels.

Since some organs in the abdomen in CT images are similar in shape and texture to the kidney, they will also segment them at the end, so it is necessary to coarse segment and extracts the kidney ROI. Coarse segmentation based on FR2PAttU-Net is performed on each slice, thus constructing a 2D segmentation of kidney tumors. The model is trained from CT images with an original size of 512 × 512 pixels. The tumor and the kidney are regarded as the same type to make a label to construct a binary segmentation model. That is, the label only includes the background and the kidney. After the model segmented the tumor and kidney area, the ROI area smaller than 128 × 128 was expanded to 128 × 128 and expanded the ROI area larger than 128 × 128 to 256 × 256, it was better to obtain the kidney, tumor, and background information. Through the coarse segmentation of the kidney, the kidney region is separated, which reduces the scope of the problem and increases the chance of successful segmentation of kidney tumors. **Figure 6** shows coarse segmentation results of CT images ranging from 512 × 512 pixels to 128 × 128 pixels.

#### Fine Segmentation Based on FR2PAttU-Net Model

**Figure 7:**
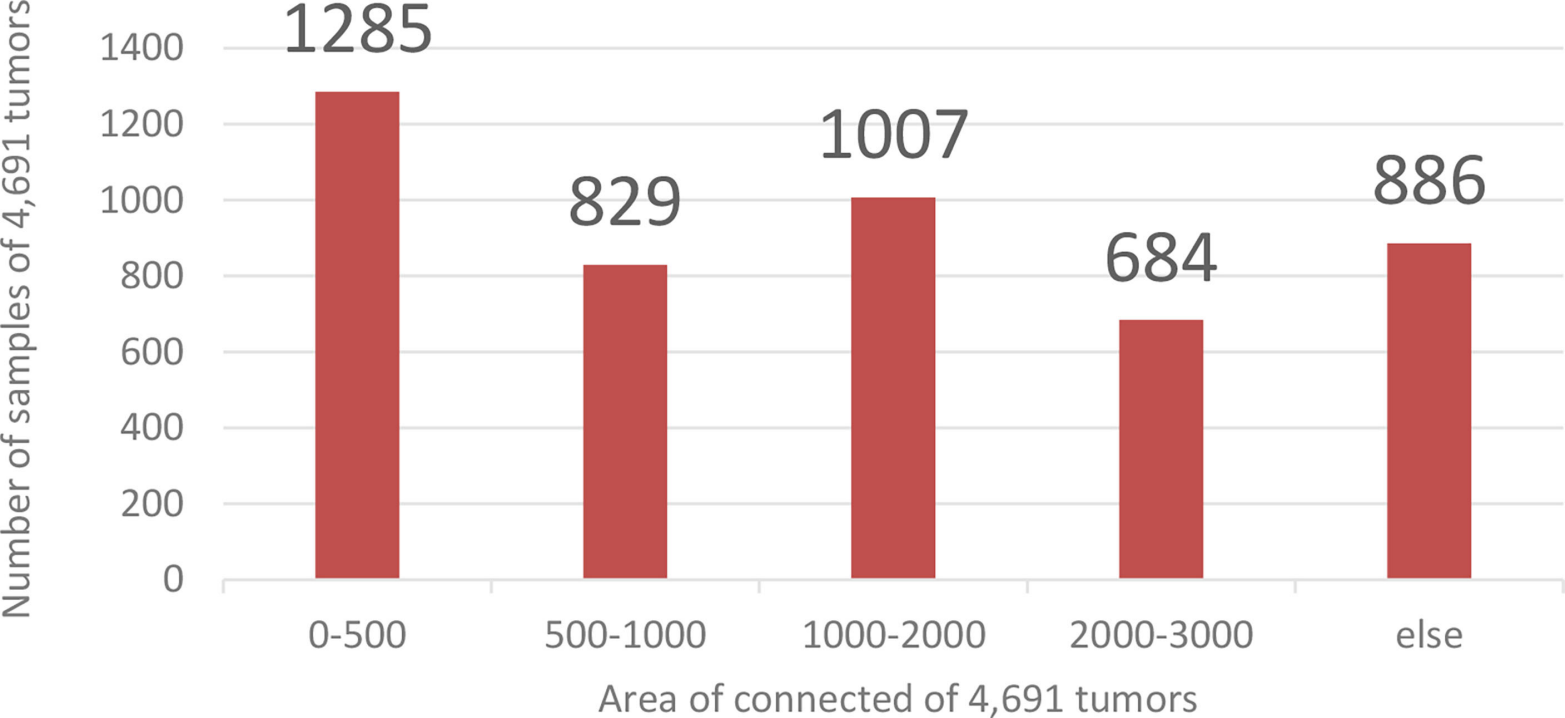
Training data distribution. The abscissa is the Area of connection of 4,691 tumors, and the ordinate is the number of samples of 4,691 tumors.

Coarse segmentation can reduce the range of the segmented image and save the entire computing resources of the model. Since the fine segmentation needs to use the coarsely segmented ROI area as training data, to avoid the impact of the imbalanced distribution of the data in training set on the tumor segmentation results, this paper needs to enhance the small sample data to balance the sample data set. This paper calculates and counts the tumor size in the training set. There are 4,691 ROI images containing tumors. The area size distribution of the connected regions is shown in **Figure 7**.

**Figure 8:**
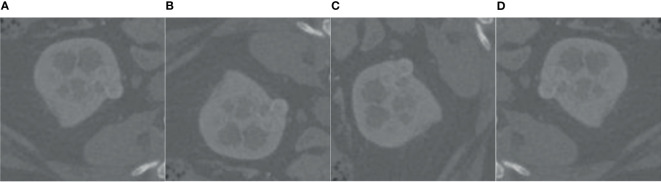
Data enhancement. **(A)** is the original kidney ROI image, **(B)** is the result of horizontal flipping, **(C)** is the result of vertical flipping, and **(D)** is the result of rotating.

Analyzing the data in **Figure 7**, we found that the tumor size distribution in the training set was not even, where the tumor area differed by about a factor of 2 between 0–500 and 2,000–3,000. Therefore, we must reconstruct the data to balance the kidney tumor segmentation dataset. For fewer datasets, we adopted data augmentation methods such as flipping, rotating, shifting, and mirroring and extended them to more data to balance the kidney tumor dataset. **Figure 8** shows several commonly used data augmentation functions.

We use the second model to accurately segment kidneys and tumors after balancing the dataset in the ROI region. Here, the input image is the kidney ROI region, all pixels predicted to be background are set to 0, and kidney and tumor are represented by different pixels.

## Experimental Results

In this section, we detail the experimental results validating the proposed method. First, we introduce the metrics used for performance validation and then discuss the results obtained in each step of the proposed process in detail. In addition to this, we also provide a series of case studies and a comparative analysis with the relevant literature.

### Evaluation Indicators

**Figure 9:**
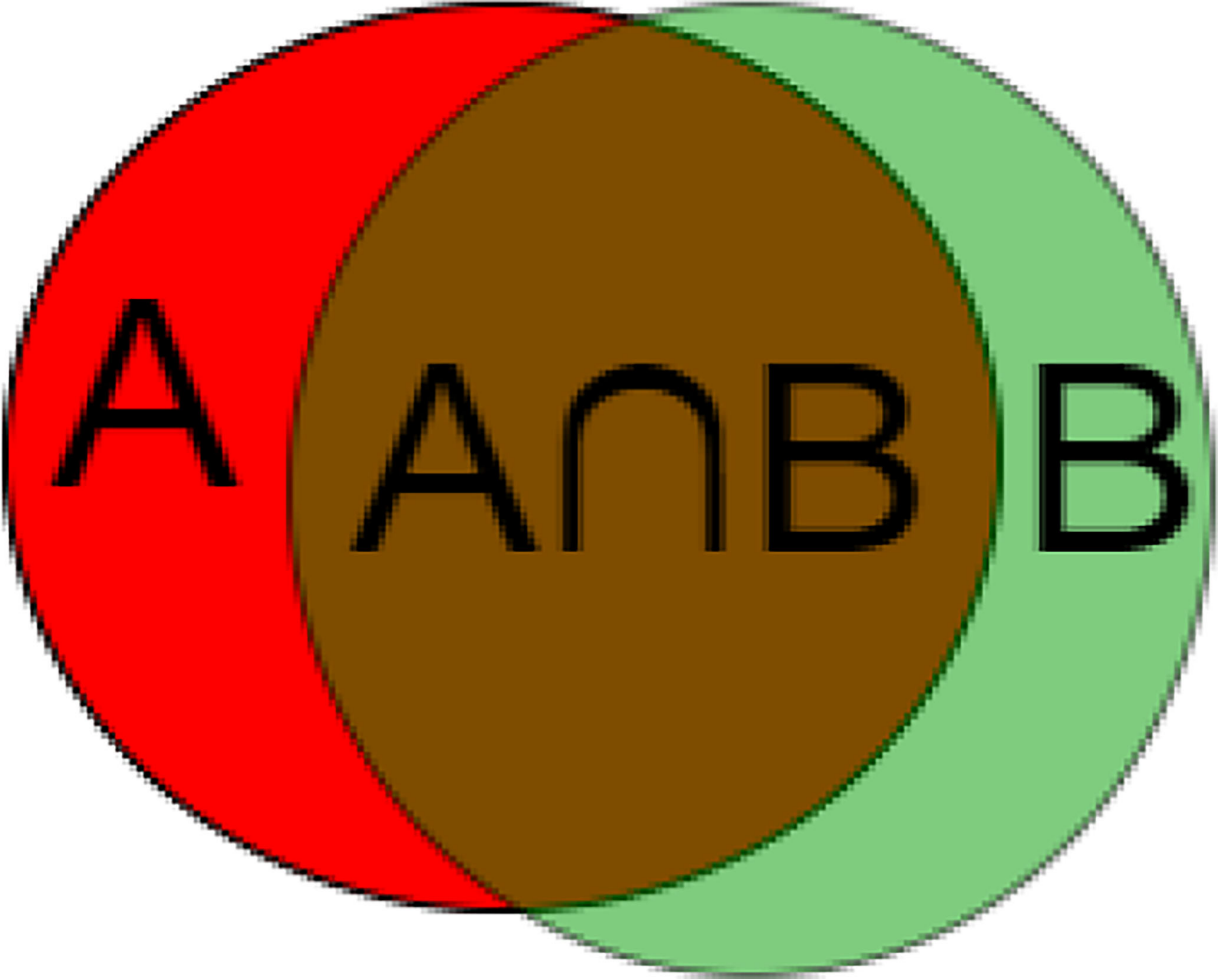
Calculation method of DSC. **(A)** segmentation result, **(B)** label.

To measure the accuracy of our method, we use metrics commonly used in CAD/CADx systems to evaluate the classification and segmentation methods of medical images (30). The metric used is the Dice similarity coefficient. It measures the spatial similarity or overlap between two segments and is commonly used to evaluate the ground truth and segmentation performance of the medical images. Equation (4) and **Figure 9** shows the calculation method of DSC.


(4) $$\bar{DSC}=\frac{1}{n}\Sigma_{i=1}^{n}\frac{2|A_{i}\cap B_{i}|}{\left|A_{i}|+|B_{i}\right|}i=1,\ldots 2,n$$

This article randomly selected 200 CT images for testing, and the rest was used as the training set. To avoid that a particular image area is equal to 0 and cannot calculate the formula, we add 1 to the numerator and denominator of the calculation formula (4). Therefore, the Dice calculation method is changed to Equation (5):


(5) $$\bar{Kidney(Tumor)\;Dice}=\frac{1}{200}\sum_{i=0}^{199}\frac{2|A_{i}\cap B_{i}|+1}{|A_{i}|+|B_{i}|+1}i=0,1,2\ldots ,199$$

Among them, A*_i_* is the *i*-th segmented area, and B*_i_* is the *i*-th label image, $\bar{Kidney(Tumor)\;Dice}$ is the average of n results.

### Experimental Results

In our experiments, we used the CT data described in *Data Preparation*. Our model is trained by an Adam optimizer, and the learning coefficient is set to 0.001. The batch size is set to 8 and the total epoch is formed to 500,000 (steps_per_epoch = 500, epochs = 100). This model is trained on NVIDIA GeForce RTX 3060 (12GB) graphics processing unit (GPU).

**Figure 10:**
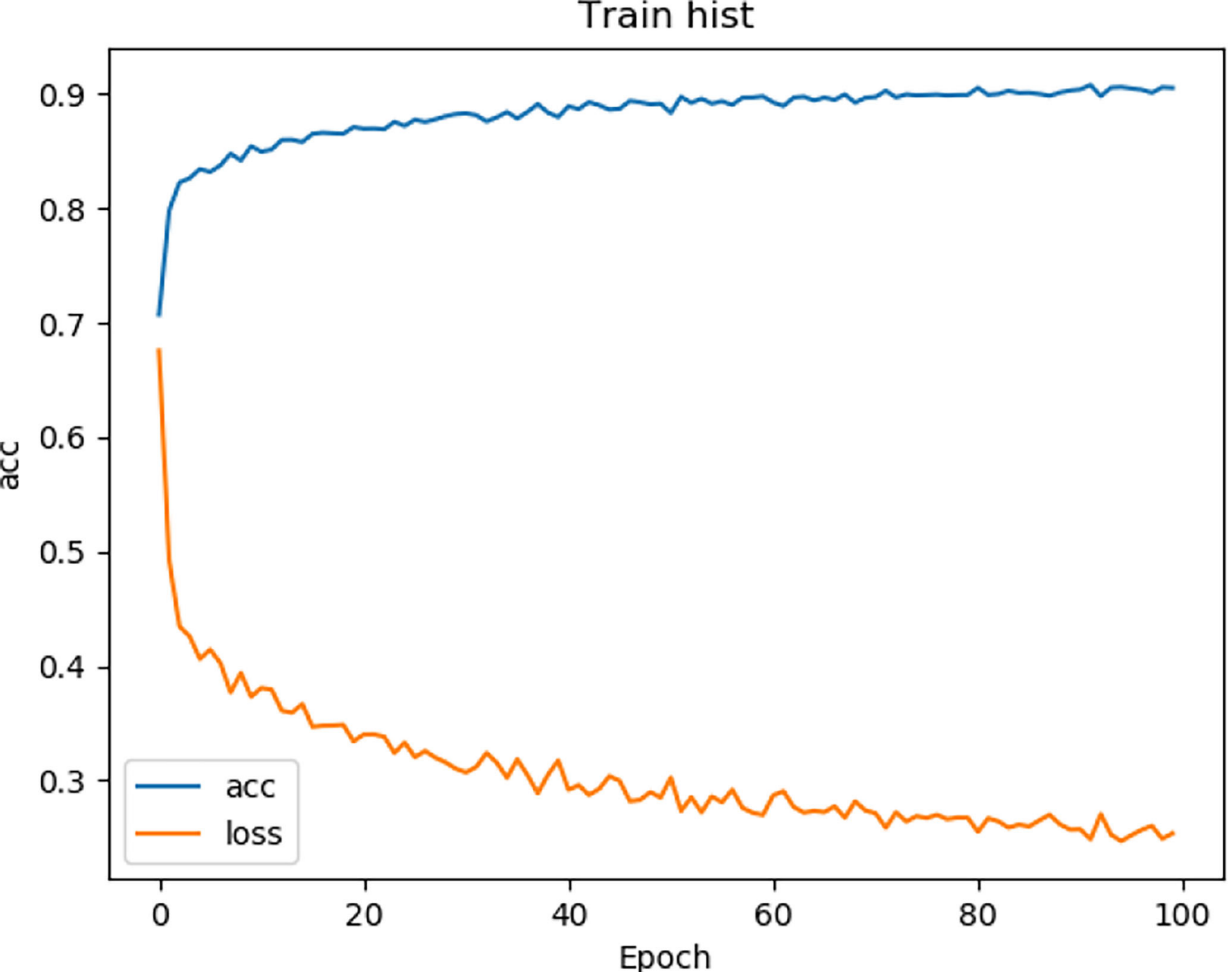
Training result (U-Net).

**Figure 11:**
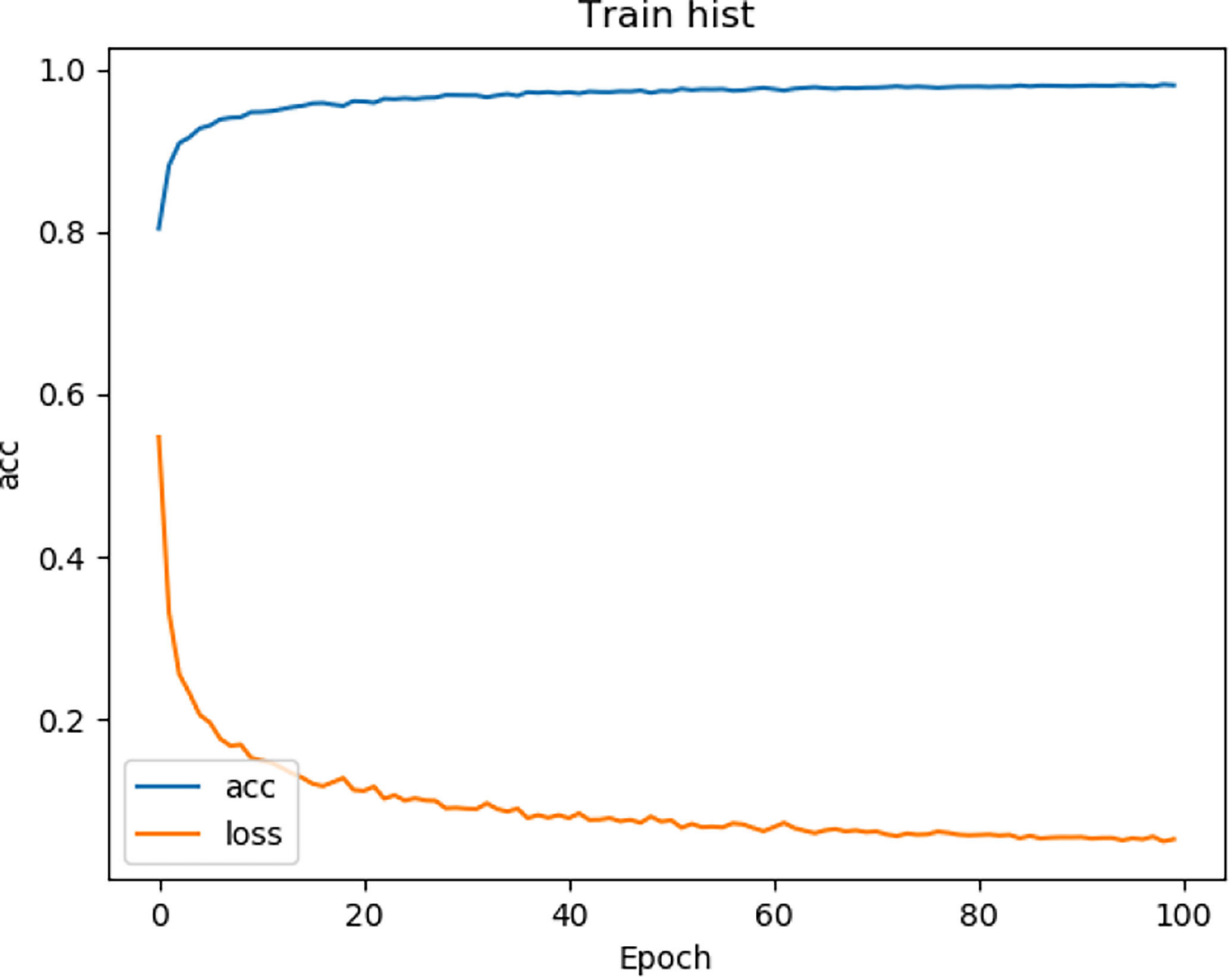
Training result (R2AttU-Net).

**Figure 12:**
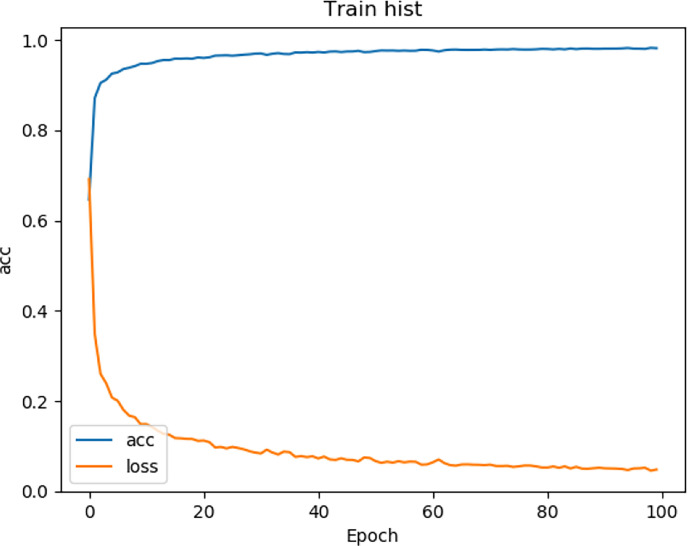
Training result [FR2PAttU-Net (kernel = 3)].

**Figure 13:**
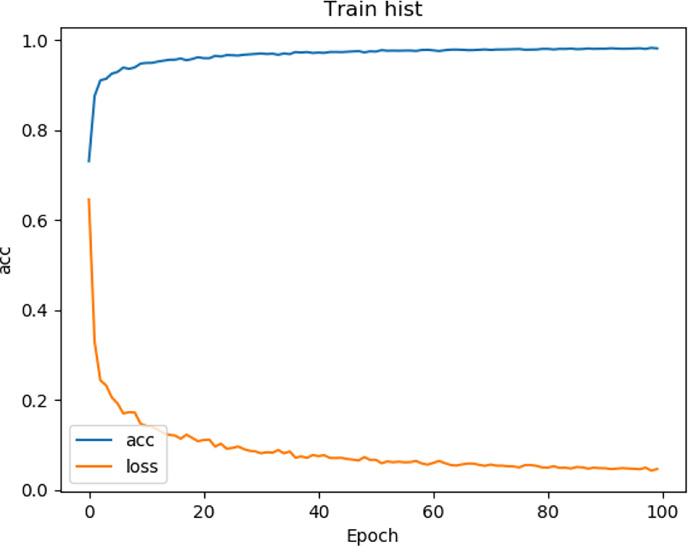
Training result [FR2PAttU-Net (kernel = 3 and 5)].

We tested the renal tumor segmentation results of multiple models on the same dataset to verify the effectiveness of the FR2PAttU-Net model for image segmentation. The U-Net model training and segmentation results are saved in **Figure 10** and **Table 1**, and the R2AttU-Net model training and segmentation results are saved in **Figure 11** and **Table 2**. **Figures 12**, **13** are the training results of FR2PAttU-Net using various convolutions, and **Tables 3**, **4** are the segmentation results of FR2PAttU-Net using various convolutions.

**Table 1:** Fine segmentation based on U-Net model.

Input image size (pixel)	Last layer image size (pixel)	Total training time	Kidney Dice	Tumor Dice	Composite score
128 × 128	8 × 8	About 500 s	0.391	0.456	0.424
128 × 128	4 × 4	About 700 s	0.472	0.415	0.444
128 × 128	2 × 2	About 1,100 s	0.583	0.460	0.522
Average			0.482	0.444	0.463

**Table 2:** Fine segmentation based on R2AttU-Net model.

Input image size (pixel)	Last layer image size (pixel)	Total training time	Kidney Dice	Tumor Dice	Composite score
128 × 128	8 × 8	About 1,500 s	0.906	0.836	0.871
128 × 128	4 × 4	About 2,000 s	0.925	0.858	0.892
128 × 128	2 × 2	About 3,700 s	0.921	0.867	0.894
Average			0.917	0.854	0.886

**Table 3:** Fine segmentation based on FR2PAttU-Net model (parallel convolutions [convolution kernel = 3 × 3)].

Input image size (pixel)	Last layer image size (pixel)	Total training time	Kidney Dice	Tumor Dice	Composite score
128 × 128	8 × 8	About 2,200 s	0.948	0.906	0.927
128 × 128	4 × 4	About 3,000 s	0.929	0.902	0.916
128 × 128	2 × 2	About 6,300 s	0.951	0.915	0.933
Average			0.943	0.908	0.926

**Table 4:** Fine segmentation based on FR2PAttU-Net model [parallel convolutions (convolution kernel = 3 × 3 and 5 × 5)].

Input image size (pixel)	Last layer image size (pixel)	Total training time	Kidney Dice	Tumor Dice	Composite score
128 × 128	8 × 8	About 2,700 s	0.948	0.914	0.931
128 × 128	4 × 4	About 4,400 s	0.951	0.913	0.932
128 × 128	2 × 2	About 11,400 s	0.946	0.905	0.926
Average			0.948	0.911	0.930

**Tables 1**–**4** are results of fine segmentation. That is, the input image is 128 × 128. Each table has six columns, input image pixel size, last layer image pixel size, total training time, kidney Dice, tumor Dice, and Composite score. With the deepening of the network, the image pixels of the previous layer gradually decrease until the GPU Terminates the experiment when out of memory is displayed. Comparing **Tables 1**–**4**, we find that with the deepening of the model, the training time of the model will be longer and longer, but our model can still extract better feature information. Furthermore, performing multiple convolution operations on the image in parallel can obtain different information about the input image than consecutive convolution operations; processing these operations in parallel and combining all the results will result in better image representation, resulting in a better tumor segmentation.

**Figure 14:**
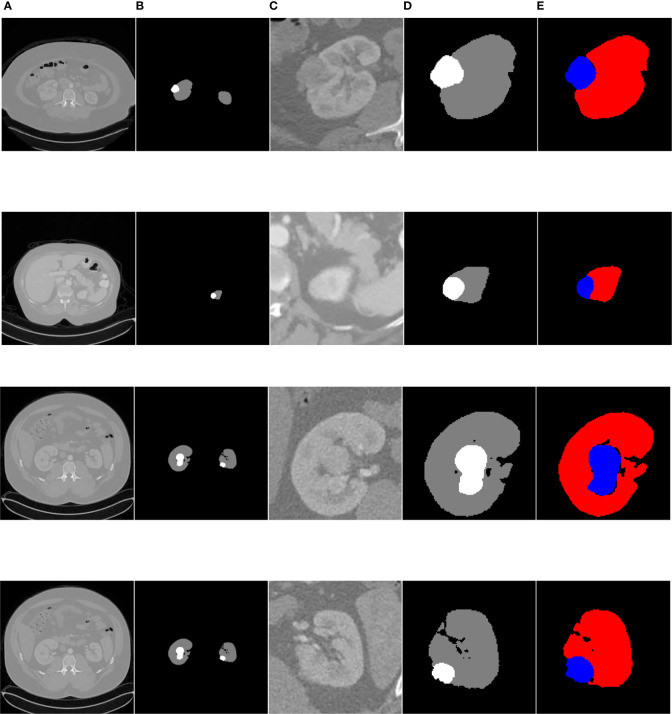
Kidney tumor segmentation based on FR2PAttU-Net model. **(A)** Original image, **(B)** Label, **(C)** Result of coarse segmentation, **(D)** label of ROI, **(E)** Result of fine segmentation.

**Figure 14** shows the overall segmentation effect based on the FR2PAttU-Net model (convolution kernel = 3 × 3 and 5 × 5) on the kidney CT images of three patients. Each patient shows five pictures, among which, A is the original image, B is the label, C is the coarse segmentation result, D is the label of ROI, and E is the fine segmentation result. **Figure 14-1** is the first type of case; the tumor and kidney are more prominent, a relatively common type. **Figure 14-2** shows the results of the second type of case. In this case, both the kidney and tumor area are small, and the tumor is blurred, making it difficult to distinguish with the human eye directly. Finally, **Figures 14-3**, **14-4** are the third types of cases in which both kidneys have tumors, our model detects two tumors separately, and two ROI regions are extracted from the image.

**Figure 15:**
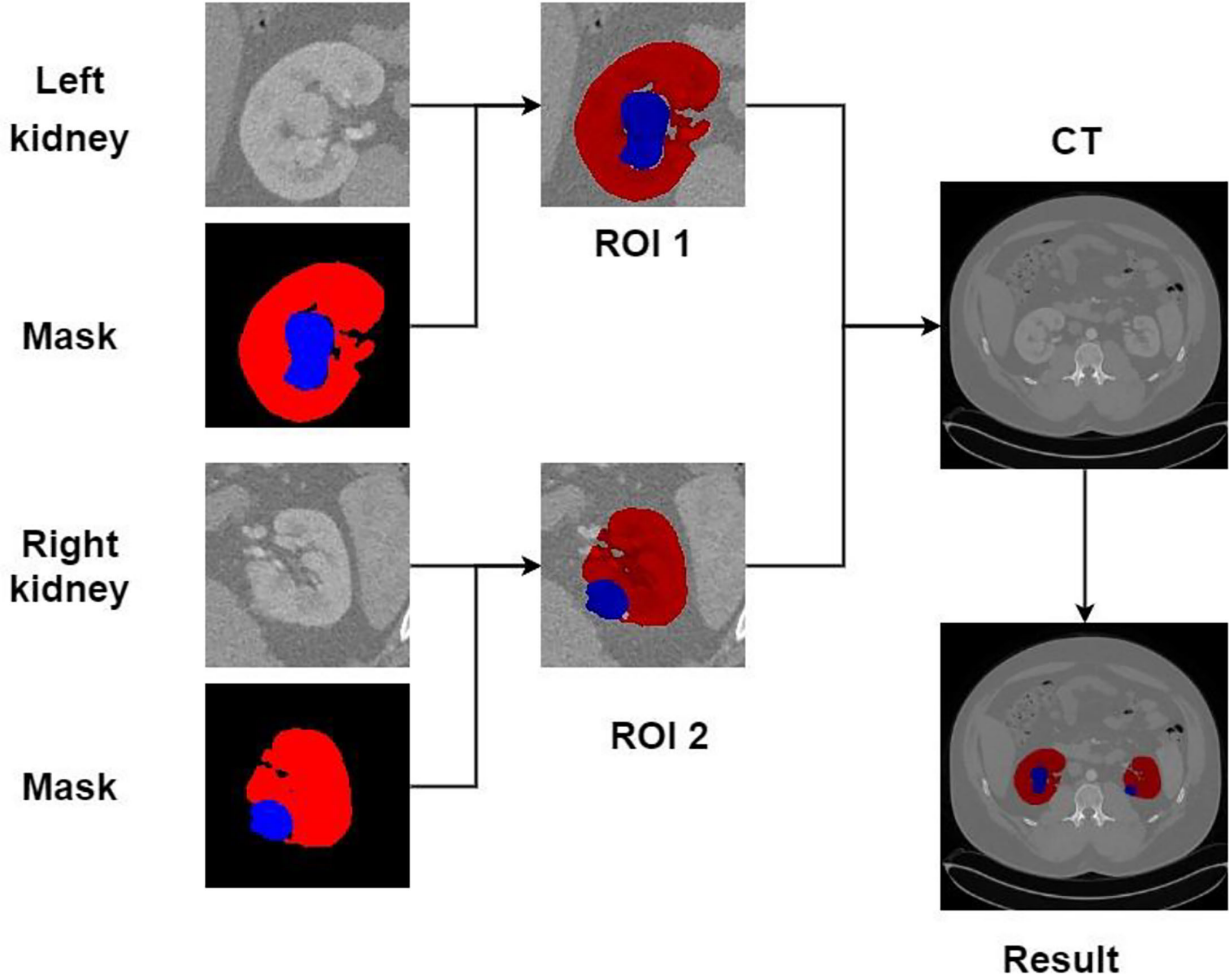
Post-processing.

Recreating the anatomy of the patient in CT images is a significant problem (31). We can post-process the CT image of the patient after the kidney tumor segmentation is completed so that the doctor can observe the spatial structure of the kidney and tumor of the patient. **Figure 15** shows the post-processing process. In the fine segmentation stage, we use an image of 128 × 128 pixels, so the segmentation result is also 128 × 128 pixels. We constructed a marked ROI region for the segmentation results of kidney and tumor (ROI 1, ROI 2). The background pixels remained unchanged and converted the pixels of the kidney and tumor into pixels of the segmentation result. The ROI area is then matched to the CT image of the patient (512 × 512 pixels), showing the specific location of the kidney and tumor of the patient, which is convenient for expert diagnosis and observation.

## Discussion and Conclusions

Many deep learning methods have been used for kidney and tumor segmentation in the past few years. **Figure 14** can intuitively see that the FR2PAttU-Net model proposed in this paper is used for the segmentation effect of kidneys and tumors. **Table 5** shows the average Dice calculated by some algorithms or methods. Among them, the data used by FR2PAttU-Net, U-Net, ResU-Net, AttU-Net, R2U-Net, R2AttU-Net, and nnU-Net are precisely the same. It is the data introduced in *Data Preparation*, and the methods of data preprocessing and data enhancement are the same. The other models from References (17, 18) and (20–24) use the KiTS19 dataset, but the FR2PAttU-Net model uses fuzzy sets to enhance the image. Therefore, we directly quoted their results without additionally testing the performance of our data on their model. As a result, we get scored a 0.948 kidney Dice and a 0.911 tumor Dice resulting in a 0.930 composite score; in the case of this test, the effect is better than U-Net, ResU-Net, AttU-Net, R2U-Net, R2AttU-Net, nnU-Net. However, our kidney Dice is about 0.2 lower when compared to other algorithms. Still, tumor Dice is about 0.4 higher, which means that the proposed method can simultaneously pay attention to the more prominent feature (kidney) and more minor features (tumors). It proves that the parallel convolution method has a particular segmentation effect and research value in kidney and tumor segmentation.

**Table 5:** Segmentation results of several algorithms or methods.

References	Algorithms or methods	Kidney Dice	Tumor Dice	Composite score
This paper	FR2PAttU-Net	0.948	0.911	0.930
Reference (11)	U-Net	0.482	0.444	0.463
Reference (12)	ResU-Net	0.688	0.694	0.691
Reference (13)	AttU-Net	0.789	0.735	0.763
Reference (14)	R2U-Net	0.681	0.711	0.696
Reference (15)	R2AttU-Net	0.917	0.854	0.886
Reference (16)	nnU-Net	0.905	0.864	0.882
Reference (17)	AlexNet+ U-Net	0.9303	\	0.9303
Reference (18)	Hybrid V-Net	0.977	0.865	0.921
Reference (20)	Cascaded U-Net ensembles	0.973	0.825	0.899
Reference (21)	Cascaded volumetric convolutional network	0.974	0.831	0.902
Reference (22)	multi-resolution VB-nets	0.973	0.832	0.903
Reference (23)	Cascaded semantic segmentation	0.967	0.845	0.906
Reference (24)	3d U-net based on five-fold cross-validation	0.974	0.851	0.912

In conclusion, this paper proposes a kidney tumor segmentation model based on FR2PAttU-Net, which can effectively segment kidney tumors. This method is a cascade deep learning model, adding residual-recurrent-parallel convolutional networks, attention gates, Leaky-ReLU, and a 20% batch normalization layer to the original U-shaped structure of the U-Net. We also use an Image enhancement algorithm with fuzzy sets to alter the input image pixels to improve the robustness of the model. The FR2PAttU-Net model increases the width of the model and enhances the adaptability of the model to the features of different image scales, and obtains an excellent segmentation effect in the kidney CT image. In future work, we will collect more medical data for validating the reliability of the FR2PAttU-Net model.

## Data Availability Statement

The raw data supporting the conclusions of this article will be made available by the authors, without undue reservation.

## Author Contributions

Data curation, ZM and TM. Formal analysis, FH and YZ. Investigation, FL. Methodology, PS. Validation, ZC. All authors listed have made a substantial, direct, and intellectual contribution to the work and approved it for publication.

## Funding

This work was supported in part by the National Natural Science Foundation of China (81873913), the National Major Instrument Development Project (61627807), and the Science and Technology Major Project of Guangxi (2019AA12005).

## Conflict of Interest

The authors declare that the research was conducted in the absence of any commercial or financial relationships that could be construed as a potential conflict of interest.

## Publisher’s Note

All claims expressed in this article are solely those of the authors and do not necessarily represent those of their affiliated organizations, or those of the publisher, the editors and the reviewers. Any product that may be evaluated in this article, or claim that may be made by its manufacturer, is not guaranteed or endorsed by the publisher.
